# Bright Edge Sign on High b-Value Diffusion-Weighted Imaging as a New Imaging Biomarker to Predict Poor Prognosis in Glioma Patients: A Retrospective Pilot Study

**DOI:** 10.3389/fonc.2019.00424

**Published:** 2019-05-21

**Authors:** Qiang Zeng, Biao Jiang, Feina Shi, Chenhan Ling, Fei Dong, Jianmin Zhang

**Affiliations:** ^1^Department of Neurosurgery, The Second Affiliated Hospital, Zhejiang University School of Medicine, Hangzhou, China; ^2^Department of Radiology, The Second Affiliated Hospital, Zhejiang University School of Medicine, Hangzhou, China; ^3^Department of Neurology, The Second Affiliated Hospital, Zhejiang University School of Medicine, Hangzhou, China; ^4^Brain Research Institute, Zhejiang University, Hangzhou, China; ^5^Collaborative Innovation Center for Brain Science, Zhejiang University, Hangzhou, China

**Keywords:** diffusion magnetic resonance imaging, glioma, prognosis, magnetic resonance imaging, imaging biomarker

## Abstract

**Purpose:** To investigate the prognostic value of bright edge sign observed on high b-value diffusion-weighted imaging (DWI) map in glioma patients.

**Methods:** We retrospectively reviewed our prospectively collected database for gliomas. Bright edge sign was defined as the presence of extremely high signal in tumor margin on high b-value DWI map (*b* = 3,000 s/mm^2^) with the signal intensity higher than those in contralateral normal white matter and tumor central region. Extremely poor prognosis was defined as overall survival time < 9 months. Survival analyses were conducted by using the Cox regression for both the univariable and multivariable analyses.

**Results:** A total of 52 patients were enrolled (WHO IV, 25; WHO III, 13; WHO II, 14). Bright edge sign presented in 10 (19.2%) patients (WHO IV, 5; WHO III, 3; WHO II, 2). Nine (90.0%) patients with bright edge sign had extremely poor prognosis, while only 1 (2.4 %) patient without bright edge sign had extremely poor prognosis. The sensitivity and specificity of bright edge sign in determining extremely poor prognosis were 90 and 97.7%, respectively. Bright edge sign (HR [95% CI] = 25.11 [7.26–86.81], *p* < 0.001) was an independent predictor of poor prognosis after adjustment.

**Conclusion:** Bright edge sign on high b-value DWI may be an accurate predictor of extremely poor prognosis in glioma patients, regardless of pathologic grades.

## Introduction

Glioma is the most common malignant tumor in the brain, accounting for about 80% of all primary malignant tumor (1). Although treatment for gliomas has been evolved, some patients still had very poor prognosis. Glioblastoma (GBM) is the most common type of gliomas with a median survival time of about 15 months. Besides, some patients with lower grade gliomas (LGG) (WHO grade II and III) also have a poor prognosis. Predicting the prognosis of patients is critical for neurosurgeons to make individualized treatment plan.

Diffusion-weighted imaging (DWI) is a convenient and non-invasive method for detecting the diffusion motion of water molecules in tissues, which has been widely used in clinical practice. DWI has been found to be effective in evaluating the pathologic grade, tumor cell density, proliferation index, and prognosis of gliomas (2–6). In clinical practice, DWI is typically obtained with a b-value of 1,000 s/mm^2^, while high b-value DWI has been found to be more useful in evaluating gliomas in many aspects (7–12).

One of our previous study has found that glioma infiltration sign on high b-value DWI can be a predictor of poor prognosis in glioma patients (13). Recently, we observed another special manifestation of extremely high signal intensity in the tumor margin on high b-value DWI maps in some glioma patients, which was termed bright edge sign. One previous study has indicated that signal intensity on high b-value DWI may be able to directly reflect water diffusivity (13). According to previous studies, extremely restricted diffusion is related to high tumor cell density and high proliferation. Thus, the presence of extremely restricted diffusion in tumor margin may indicate that the tumor is aggressive and tend to invade into peritumoral region. Moreover, central regions of tumors are easy to be removed by surgery, while peripheral tumor regions may be not removed completely. Hence, we hypothesized that the presence of bright edge sign in glioma patients might indicate a poor outcome. In this study, we aimed to investigate the association between bright edge sign and prognosis in glioma patients.

## Methods and Materials

### Patients

This retrospective study was based on our prospectively collected database for consecutive patients with gliomas who were hospitalized at our center between August 2013 and January 2015. This study was approved by the ethics review board at the Second Affiliated Hospital of Zhejiang University School of Medicine, and all the patients provided written informed consent. This study was conducted according to the principles expressed in the Declaration of Helsinki. This study enrolled patients who (1) age > 18 years; (2) underwent preoperative MRI examination with a multi-b-value DWI sequence; (3) underwent tumor resection; (4) had a glioma confirmed by pathology. This study excluded patients who (1) had received chemotherapy, radiotherapy, or hormonotherapy prior to the preoperative MRI examination; (2) died of operative complications; (3) were lost to follow-up.

### Clinical Data

Preoperative Karnofsky performance score (KPS) was recorded for each patient on admission. Histopathologic diagnosis was performed by pathologists and based on the WHO criteria. Whether a patient ever received postoperative radiotherapy with or without chemotherapy was recorded. Overall survival (OS) was defined as the time from diagnosis until either death or the time the patient was last known to be alive (censored) (14). Patients were dichotomized into extremely poor prognosis group (OS time < 9 months) and relatively favorable prognosis group (OS time ≥ 9 months).

### Image Acquisition and Analysis

All subjects underwent a preoperative MRI examination on a 3.0-Tesla MR system (Discovery MR750, GE Healthcare Systems, Milwaukee, WI) with an 8-channel high-resolution receiver head coil. A DWI sequence was performed with nine b-values (0, 100, 200, 300, 500, 700, 1,000, 2,000, and 3,000 s/mm^2^) in three orthogonal directions using a single-shot echo planer imaging with the following parameters: section thickness, 4 mm; spacing between slices, 5 mm; repetition time/echo time, 3,000/88.6 ms; field of view, 240 × 240 mm; matrix, 256 × 256; and flip angle, 90. Besides, a contrast-enhanced T1-weighted sequence and a contrast-enhanced T2-flair sequence were also acquired after the injection of gadodiamide.

Bright edge sign was defined as the presence of extremely high signal in tumor margin on high b-value DWI map (*b* = 3,000 s/mm^2^) with the signal intensity higher than those in contralateral normal white matter and tumor central region. Regions with high signal intensities which were suspected as necrosis or hemorrhage were excluded from the assessment. The DWI maps and the contrast-enhanced T1-weighted images were both co-registered to the contrast-enhanced T2-flair images using SPM12 (available at www.fil.ion.ucl.ac.uk/spm), and were referred when identifying tumor margins. The bright edge sign was assessed independently by an experienced neurosurgeon with more than 30 years' experience (JZ, reader 1) and an experienced neuroradiologist with 20 years' experience (BJ, reader 2). Disagreement between these two readers was resolved by another neuroradiologist with 7 years' experience (FD, reader 3). These readers were all blinded to the patients' clinical information.

In addition, tumor volumes were calculated by multiplying voxel numbers in regions of interest by the volume of one voxel. The regions of interest were drawn slice by slice for each patient on DWI maps with *b* = 3,000 s/mm^2^ by an experienced neuroradiologist who was blinded to the tumor pathology, and necrosis areas, cystic lesions and hematomas were carefully excluded (12). Whether a glioma was across the midline was also recorded.

### Statistical Analysis

All statistical analyses were performed with SPSS Statistics, Version 22 (IBM, Armonk, New York). The kappa statistic value was used to assess interobserver concordance for the assessment of glioma infiltration sign between reader 1 and reader 2. Medians with interquartile range (IQR) and percentages were used to describe the distribution of continuous and categorical variables, respectively. Differences between two groups were estimated by Mann-Whitney *U*-test for continuous variables, and were estimated by Chi-Square test for categorical variables. Kaplan-Meier (K-M) survival curves were also generated. Survival analyses were conducted by using the Cox regression for both the univariable and multivariable analyses. Variables with a *p* < 0.2 in univariable analysis were included in multivariable analysis. A *p* < 0.05 were considered as statistically significant.

## Results

### Baseline Characteristics

Finally, a total of 52 patients were enrolled in this study (WHO IV, 25; WHO III, 13; WHO II, 14). Of these included patients, the median age was 45 (IQR, 39–57) years and 31 (59.6%) patients were male. The median KPS on admission was 90 (IQR, 80–90), and the median tumor volume was 8.5 (3.8–20.4) cm^3^.

**Figure 1:**
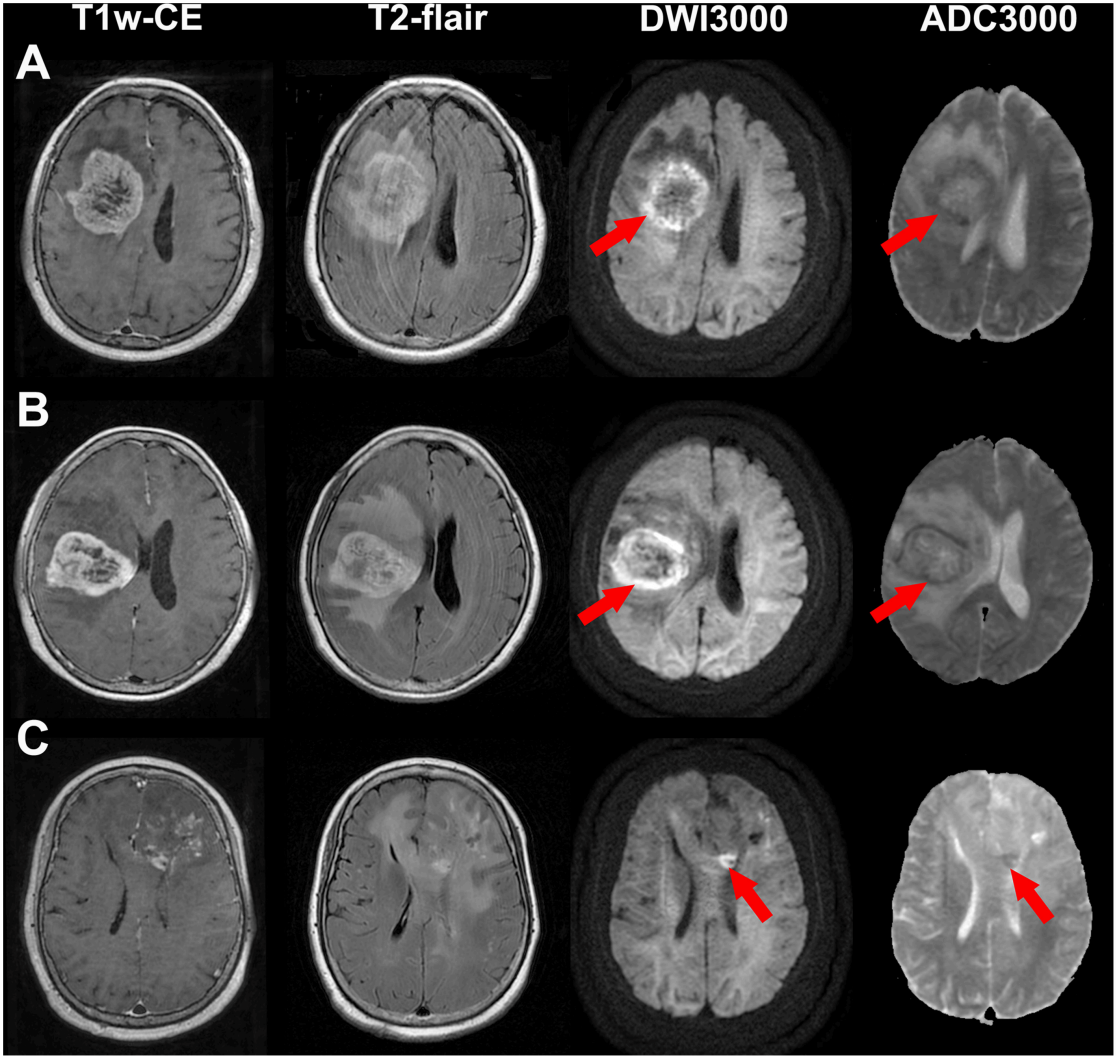
Illustrations of a glioblastoma (WHO grade IV) **(A)**, an anaplastic astrocytoma (WHO grade III) **(B)** and an astrocytoma (WHO grade II) **(C)** with bright edge sign. T1-weighted contrast-enhanced images, T2-flair contrast-enhanced images, DWI maps with *b* = 3,000 s/mm^2^ and ADC maps with *b* = 3,000 s/mm^2^ are shown in the first to forth rows, respectively. Red arrows show the extremely high signal regions in tumor margins on DWI maps. Note that the ADC values of these regions are low.

**Figure 2:**
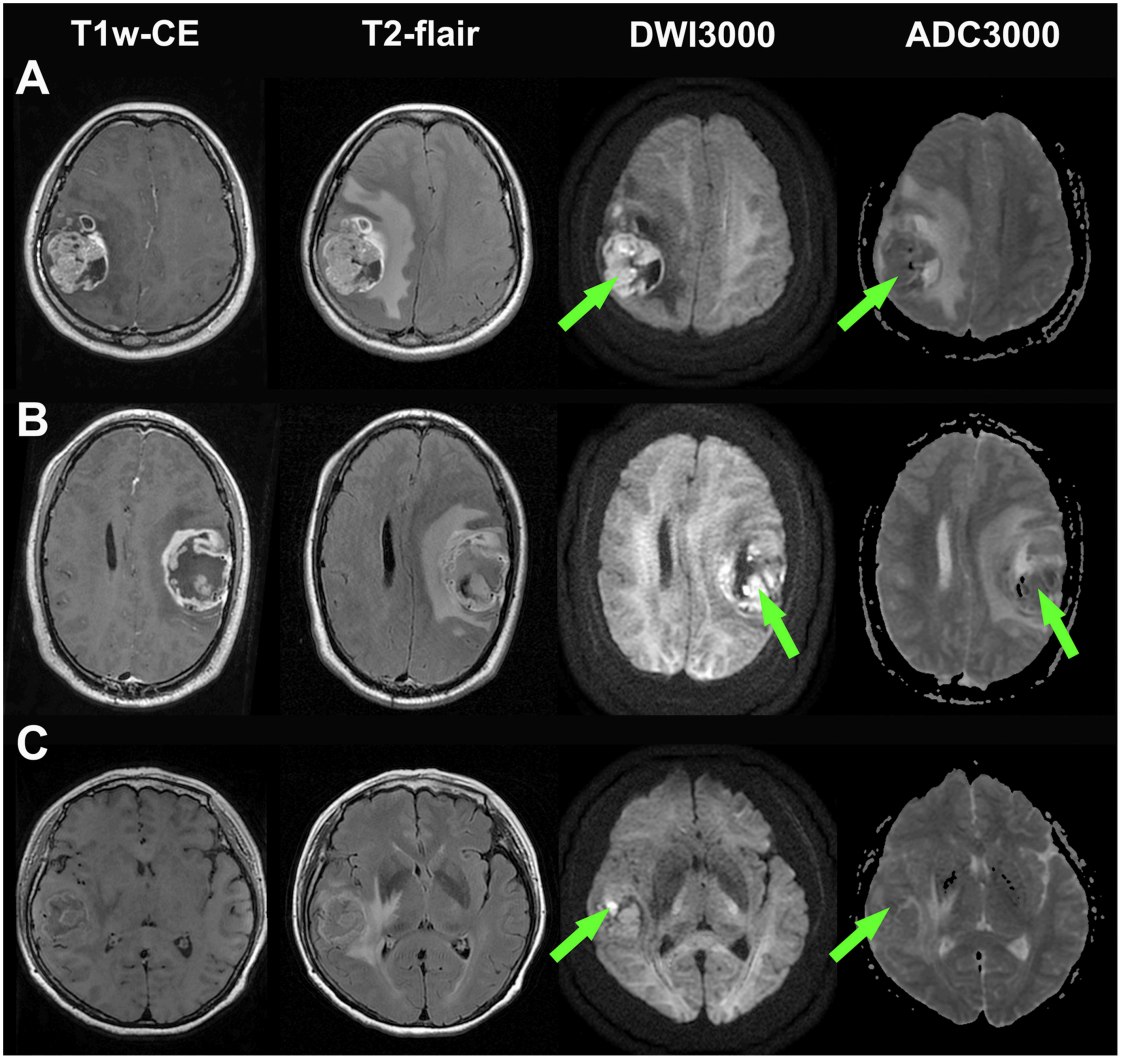
Illustrations of three glioblastomas without bright edge sign. **(A)** a glioblastoma patient who had survived for 30 months (still alive at last time follow-up), **(B)** a glioblastoma patient who had a survival time of 33 months, and **(C)** a glioblastoma patient who had a survival time of 24 months. T1-weighted contrast-enhanced images, T2-flair contrast-enhanced images, DWI maps with *b* = 3,000 s/mm^2^ and ADC maps with *b* = 3,000 s/mm^2^ are shown in the first to forth rows, respectively. Note that extremely high signal regions exist within tumor regions on DWI maps (Green arrow).

Bright edge sign was observed in 10 (19.2%) patients (WHO IV, 5; WHO III, 3; WHO II, 2). The interobserver kappa value for the assessment of bright edge sign was 0.94. Example illustrations of patients with bright edge sign and without bright edge sign are shown in Figures 1, 2, respectively. Patients with bright edge sign had a lower preoperative KPS than patients without bright edge sign (80 [50–90], 90 [90–90], respectively, *p* = 0.012), shown in Table 1. No significant differences were detected in age, gender, tumor volume, tumor grade, and the occurrence rate of across the midline between two groups (all *p* > 0.05).

**Table 1:** Baseline characteristics of patients.

**Variables**	**Total (*n* = 52)**	**Bright edge sign (*n* = 10)**	**No bright edge sign (*n* = 42)**	***p*-value**
Age, year	45 (39–57)	57 (44–63)	44 (38–56)	0.086
Male, n (%)	31 (59.6%)	5 (50.0%)	26 (61.9%)	0.741
Preoperative KPS	90 (80–90)	80 (50–90)	90 (90–90)	0.012
Across the midline, n (%)	10 (19.2%)	4 (40.0%)	6 (14.3%)	0.159
Tumor volume, cm^3^	8.5 (3.8–20.4)	13.4 (2.0–25.9)	8.2 (3.8–20.1)	0.472
Tumor Grade, *n* (%)				0.833
Grade II	14 (26.9%)	2 (20.0%)	12 (28.6%)	
Grade III	13 (25.0%)	3 (30.0%)	10 (23.8%)	
Grade IV	25 (48.1%)	5 (50.0%)	20 (47.6%)	

*KPS, Karnofsky performance score*.

### Survival Analysis

Totally, 10 patients were classified into extremely poor prognosis group. Nine (90.0%) patients with bright edge sign and only 1 (2.4%) patient without bright edge sign had extremely poor prognosis. The sensitivity and specificity for bright edge sign in determining extremely poor prognosis were 90 and 97.7%, respectively.

Particularly, for the 27 LGG patients, all 5 patients with bright edge sign had extremely poor prognosis, while all other 22 patients without bright edge sign had relatively favorable prognosis. Among these 22 LGG patients without bright edge sign, 1 patient (WHO grade III) had an OS time of 22 months, 1 patient (WHO grade III) had an OS time of 42 months, and other 20 patients were still alive at last follow-up (> 36 months).

**Figure 3:**
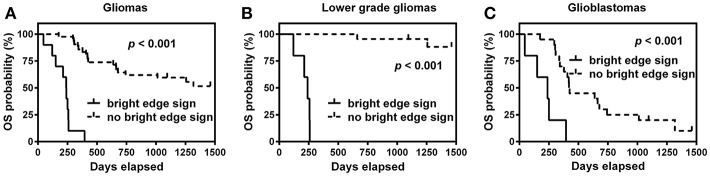
Kaplan-Meier survival curves of overall survival (OS) for patients with or without bright edge sign in all gliomas **(A)**, lower grade gliomas **(B)**, and glioblastomas **(C)**.

K-M survival curves are shown in Figure 3. The median OS time was only 8.0 months for patients with bright edge sign, while it could not be estimated for patients without bright edge sign because less than half of these patients were died. The log-rank test showed that the presence of glioma infiltration sign was significantly associated with poor OS in patients with all glioma, LGG or GBM (all *p* < 0.001).

Univariable Cox regression analysis showed that bright edge sign was significantly associated with poor OS (HR [95% CI] = 22.48 [7.85–64.40], *p* < 0.001), shown in Table 2. Elder age, lower preoperative KPS and higher tumor grade were also significantly associated with poor OS (all *p* < 0.01). Patients with tumor across the midline and not received post-operative treatment tended to have a poor OS (*p* = 0.063 and 0.102, respectively). Gender and tumor volume were not associated with OS (*p* = 0.701 and 0.235, respectively). Multivariable Cox regression analysis showed that bright edge sign could independently predict poor OS (HR [95% CI] = 25.11 [7.26–86.81], *p* < 0.001) after adjustment for age, preoperative KPS, across the midline, tumor grade, and postoperative treatment, shown in Table 2.

**Table 2:** Univariable and multivariable Cox model of variables in predicting overall survival in glioma patients.

	**Univariable analysis**			**Multivariable analysis**		
**Variables**	**HR**	**95% CI**	***p*-value**	**HR**	**95% CI**	***p*-value**
Age	1.04	1.01–1.07	0.006	1.05	1.01–1.08	0.005
Gender (Male vs. Female)	0.86	0.41–1.83	0.701			
Preoperative KPS	0.97	0.95–0.99	< 0.001	1.00	0.98–1.03	0.763
Across the midline vs. not	2.18	0.96–4.95	0.063	2.32	0.77–7.02	0.135
Tumor volume	1.02	0.99–1.05	0.235			
Tumor grade	3.47	1.83–6.58	< 0.001	6.00	2.45–14.70	< 0.001
Postoperative treatment vs. not	0.52	0.23–1.14	0.102	0.24	0.08–0.66	0.006
Bright edge sign vs. not	22.48	7.85–64.40	< 0.001	25.11	7.26–86.81	< 0.001

*KPS, Karnofsky performance score*.

## Discussion

Predicting prognosis of gliomas is critical for individualized treatment plan in clinical practice. In our retrospective cohort, we found that bright edge sign on high b-value DWI could predict extremely poor prognosis in gliomas. Particularly, all five LGG patients with bright edge sign had extremely poor prognosis, indicating these patients may need more aggressive treatment regardless of pathological grade, especially for WHO grade II gliomas.

DWI is routinely performed in glioma patients [C. (15)]. In clinical practice, DWI maps are typically obtained with *b* = 1,000 s/mm^2^. Due to T2 shine-through effect on DWI maps, doctors need refer to ADC maps to verify whether a high signal region on DWI map is truly diffusion-limited (16). However, T2 shine-through effect reduces with increasing *b* value (7, 17, 18), making it possible for DWI maps obtained with high b-value to directly reflect water diffusion (13). Seo et al. found that high *b*-value DWI maps was solely effective in grading gliomas without referring to ADC maps (7). On DWI maps with *b* = 3,000 s/mm^2^, most high-grade gliomas (WHO grade III–IV) are hyperintense, and most low-grade gliomas (WHO grade I–II) are isointense or hypointense (7).

There are several potential mechanisms to explain that patients with bright edge sign have extremely poor prognosis. First, extremely high signal in tumor margin on high b-value DWI maps and relatively low signal in tumor central regions may indicate that the tumor has an invasive growth pattern and tends to invade into peritumoral regions, leading to an extremely poor prognosis. Secondly, although intraoperative neuronavigation system and 5-ALA fluorescence image guided resection can aid to achieve gross-total resection (19), peripheral regions with tumor cell infiltration may be not removed completely. Residual tumor cell with high proliferation activity may grow back at a short time in patients with bright edge sign. Thus, removing the regions with ‘bright edge' in tumor margin during surgery may contribute to a better prognosis. In addition, it is also doubtable whether gene phenotype differ between patients with and without bright edge sign. The exact mechanisms should be studied in further studies.

In our study, it is interesting to find that two WHO grade II gliomas with bright edge sign also had an OS time < 9 months while other WHO grade II gliomas were all still alive at last follow-up (OS time > 36 months). Some other potential mechanisms should be considered further. High-grade gliomas always contain both low- and high-grade components, and sampling error may occur in pathological assessment (20, 21). It is doubtful that whether these two low grade gliomas with bright edge sign contained high-grade component in the extremely high signal regions. Thus, tumor sampling guided by high b-value DWI may be needed for these patients when undergo surgery to ensure a correct pathologic grade. In addition, these patients may need adjuvant chemoradiotherapy after surgery regardless of tumor grade.

One of our previous study has proposed a glioma infiltration sign on high b-value DWI maps as an independent predictor of poor prognosis in glioma patients (13). The glioma infiltration sign was defined as the presence of peritumoral abnormal high signal region on a high *b*-value DWI map, which was adjacent to the tumor region and had obviously higher signal than surrounding peritumoral areas (13). This sign was suspected as a manifestation of tumor infiltration. Thus, although the glioma infiltration sign and bright edge sign are both observed on high b-value DWI, the definitions and meanings of these two signs are totally different. Besides, the presence of glioma infiltration sign was found to be highly correlated with tumor grade (13), while the presence of bright edge sign was not associated with tumor grade.

There are several limitations in this study. First, this is a retrospective study, and inherent limitations exist in this kind of studies, such as selection bias. Second, the sample size is relative small. Third, molecular pathology, which is very important for gliomas, was not routinely examined in these patients. Further well-designed prospective studies enrolling larger samples are needed to verify the prognostic value of bright edge sign and investigate the related mechanisms.

In conclusion, we presented a bright edge sign on high b-value DWI maps in glioma patients. Our findings demonstrate that bright edge sign is a useful imaging marker of poor prognosis regardless of tumor grade. Thus, patients with bright edge sign may need more aggressive treatment, especially for LGG patients. These finding can also guide further researches to find new methods to prolong survival time in these patients. High b-value DWI may be a convenient, non-invasive and useful method for evaluating gliomas, and we recommend that it can be widely applied and studied in clinical practice.

## Ethics Statement

This study was approved by the ethics review board at the Second Affiliated Hospital of Zhejiang University school of Medicine, and all the patients provided written informed consent. This study was conducted according to the principles expressed in the Declaration of Helsinki.

## Author Contributions

QZ conceived the idea and wrote the main manuscript text. QZ, CL, and FD collected the data. JZ, BJ, FD, QZ, and FS analyzed the data. All authors reviewed the manuscript.

### Conflict of Interest Statement

The authors declare that the research was conducted in the absence of any commercial or financial relationships that could be construed as a potential conflict of interest.
